# Utilizing voluntary wheel running to attenuate deficits in cerebral blood flow in the J20 mouse model

**DOI:** 10.1002/alz70855_096746

**Published:** 2025-12-23

**Authors:** Jack L Browning, Allie Kaloss, Jiangtao Li, Michelle Theus, Michelle L Olsen

**Affiliations:** ^1^ Virginia Tech, Blacksburg, VA, USA

## Abstract

**Background:**

Reduced cerebral blood flow (CBF) represents one of the earliest observable pathologies of Alzheimer's Disease (AD), and is recapitulated in animal models of AD with amyloid beta (Aβ) accumulation. Chronic reduction in CBF and the subsequent reduction in parenchymal tissue oxygenation and glucose may be sufficient to drive neurodegeneration. Our preliminary studies suggest reduced CBF may result from massive Aβ accumulation on the leptomeningeal vessels (∼40% vessel coverage in male AD mice), including the MCA and PCA, and connecting collaterals in aged mice. Aerobic exercise represents one potential therapeutic which augments CBF and serves as a functional adaptation of collateral vessels. Here, we seek to address if aerobic exercise in AD mice impacts disease progression in part by mitigating meningeal AB load and reduced CBF.

**Methods:**

Using a Methoxy‐X04 and vessel painting approach, we quantify the total burden of cerebral amyloid angiopathy (CAA) in the leptomeninges of J20 mice, an animal model of AD, and assess global CBF changes through laser speckle contrast imaging (LSCI). Further, we have assessed the impacts of long term voluntary wheel running on memory, cerebral blood flow, and leptomeningeal Aβ accumulation.

**Results:**

Leptomeningeal CAA burden is significant in AD mice relative to parenchymal Aβ accumulation in J20 mice. Further CBF is reduced ∼15% in 12‐month old J20 mice relative to WT controls, with a significant correlation between high leptomeningeal CAA burden and reduced CBF. Uninterrupted access to a free running wheel is sufficient to reduce cognitive deficits and is associate with significantly higher global CBF than sedentary controls as measured by LSCI. Ongoing studies are aimed at determining if these changes are associated with reduced leptomeningeal Aβ burden.

**Conclusion:**

These findings suggest that leptomeningeal CAA may restrict blood perfusion into the parenchyma. Further experiments will be conducted to determine whether exercise is capable of reducing leptomeningeal CAA and identifying alternative mechanisms through which exercise can restore CBF.

